# Nerolidol and Farnesol Inhibit Some Cytochrome P450 Activities but Did Not Affect Other Xenobiotic-Metabolizing Enzymes in Rat and Human Hepatic Subcellular Fractions

**DOI:** 10.3390/molecules22040509

**Published:** 2017-03-24

**Authors:** Alena Špičáková, Barbora Szotáková, Diana Dimunová, Zuzana Myslivečková, Vladimír Kubíček, Martin Ambrož, Kateřina Lněničková, Kristýna Krasulová, Pavel Anzenbacher, Lenka Skálová

**Affiliations:** 1 Department of Pharmacology and Institute of Molecular and Translational Medicine, Faculty of Medicine, Palacky University, Hněvotínská 3, 77515 Olomouc, Czech Republic; alena.spicakova@upol.cz (A.Š.); kristyna.krasulova@upol.cz (K.K.); pavel.anzenbacher@upol.cz (P.A.); 2 Department of Biochemical Sciences, Faculty of Pharmacy, Charles University, Akademika Heyrovského 1203, 50005 Hradec Králové, Czech Republic; dimunovd@faf.cuni.cz (D.D.); zuzka.mysliveckova@seznam.cz (Z.M.); ambrozm@faf.cuni.cz (M.A.); lnenickk@faf.cuni.cz (K.L.); skaloval@faf.cuni.cz (L.S.); 3 Department of Biophysics and Physical Chemistry, Faculty of Pharmacy, Charles University, Akademika Heyrovského 1203, 50005 Hradec Králové, Czech Republic; kubicek@faf.cuni.cz

**Keywords:** nerolidol, farnesol, inhibition, drug-metabolizing enzymes

## Abstract

Sesquiterpenes, 15-carbon compounds formed from three isoprenoid units, are the main components of plant essential oils. Sesquiterpenes occur in human food, but they are principally taken as components of many folk medicines and dietary supplements. The aim of our study was to test and compare the potential inhibitory effect of acyclic sesquiterpenes, *trans*-nerolidol, *cis*-nerolidol and farnesol, on the activities of the main xenobiotic-metabolizing enzymes in rat and human liver in vitro. Rat and human subcellular fractions, relatively specific substrates, corresponding coenzymes and HPLC, spectrophotometric or spectrofluorometric analysis of product formation were used. The results showed significant inhibition of cytochromes P450 (namely CYP1A, CYP2B and CYP3A subfamilies) activities by all tested sesquiterpenes in rat as well as in human hepatic microsomes. On the other hand, all tested sesquiterpenes did not significantly affect the activities of carbonyl-reducing enzymes and conjugation enzymes. The results indicate that acyclic sesquiterpenes might affect CYP1A, CYP2B and CYP3A mediated metabolism of concurrently administered drugs and other xenobiotics. The possible drug–sesquiterpene interactions should be verified in in vivo experiments.

## 1. Introduction

Sesquiterpenes, defined as 15-carbon compounds formed from three isoprenoid units, are an extremely diverse, heterogeneous and large group of natural compounds. Sesquiterpenes, together with monoterpenes, represent the major components of plant essential oils, widely used in folk medicines, health-supporting preparations and cosmetics [1]. Sesquiterpene structures form several acyclic, mono-, bi-, tri-, and tetracyclic systems. The acyclic representatives, also called farnesans, are derived directly from the basic structure, farnesol (FAR).

Farnesol occurs in, among others, cabreuva, ambrette, jasmine, ylang, and rose essential oils. FAR has a broad spectrum of biological activities including antioxidant, anti-inflammatory, anti-allergic, chemo-preventive and anticancer effects. For example, FAR ameliorates serum allergic antibody titres and lipid profiles in ovalbumin-challenged mice [2], and might have an anti-inflammatory potential to allergic asthmatic mice [3]. FAR attenuates lipopolysaccharide-induced neurodegeneration in mice by regulating intrinsic apoptotic cascade [4]. FAR is an effective inducer of cell cycle arrest and apoptosis in a variety of carcinoma cell types. In addition, FAR has been reported to inhibit tumorigenesis in several animal models, suggesting that it functions as a chemo-preventive and anti-tumor agent in vivo [5,6]. Oral FAR treatment of mice had the cardioprotective effect which was accompanied by increased protein geranylgeranylation and seemed to be independent of the antioxidant effect of FAR [7].

Nerolidol (NER) is the allylic isomer of farnesol and exists in two geometric isomers, a *trans* and a *cis* form. They can be found in cabreuva, niaouli and neroli oils, among others. NER is a common ingredient in many food and cosmetic products. In organisms, it exerts many biological effects e.g., antioxidant, antibacterial, anti-parasite and anticancer [8]. For example, NER was effective against babesiosis [9] and schistosomiasis [10]. Moreover, NER was able to improve the efficacy of drugs in treatment of malaria in mice [11]. Neuroprotective effect of NER, mediated through its anti-oxidant and anti-inflammatory activities, was observed in rats [12]. Using HeLa and Jurkat cell lines, strong anti-tumor effects of NER at concentrations less than 5 µM was found [13].

**Figure 1:**
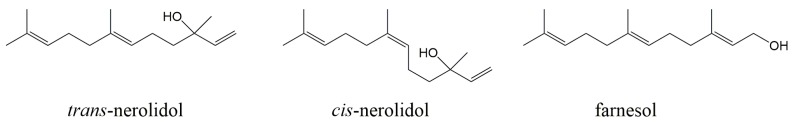
Structural formulae of used sesquiterpenes, *trans*-nerolidol, *cis*-nerolidol, and farnesol.

Taken together, both acyclic sesquiterpenes mentioned above (structures are presented in Figure 1) have interesting biological activities, and their use in human therapy might be considered. Moreover, popularity of herbal products and essential oils containing these sesquiterpenes in folk medicines increases. In spite of these facts, FAR, and particularly NER isomers, have not been properly studied for their possible herb–drug interactions. These interactions, based on the ability to modulate activities of important drug-metabolising enzymes such cytochromes P450 (CYP), carbonyl reductase 1 (CBR1), NADPH-quinone oxidoreductase 1 (NQO1), aldo-keto reductases (AKR), UDP-glucuronosyltransferases (UGT), sulfotransferases (SULT) and glutathione S-transferases (GST), may cause serious adverse effects on human health.

Cytochrome P450 (CYP)-catalyzed oxidative reactions are the most common phase I reactions in drug metabolism. However, CYPs are not the only enzymes involved in the phase I metabolism of drugs and other xenobiotics—carbonyl reduction is the major phase I reaction for an array of xenobiotics [14]. Carbonyl groups are frequently found in endogenous or xenobiotic compounds. They can promote oxidative stress, the products of which are thought to be an important initiating factor in degenerative diseases or cancer. Distinct enzymes, belonging to several families, mainly to aldo-keto reductases (AKR) and short-chain dehydrogenases/reductases, reduce carbonyl groups. These reductases often show broad and overlapping substrate specificities and some well-characterized members (e.g., CBR1, AKR1A, AKR1C, or NQO1), and have protective roles toward xenobiotic carbonyls and quinones, because metabolic reduction leads to less toxic products, which can be further conjugated with endogenous substrate and excreted [15,16,17,18].

Therefore, the present study was designed to evaluate the inhibitory effect of three abundant acyclic and biologically active sesquiterpenes farnesol, *trans*-nerolidol (TNER) and *cis*-nerolidol (CNER) on the activities of CYPs, CBR1, NQO1, AKRs, GSTs, UGTs and SULTs. With respect to possible inter-species differences in an action of inhibitors on drug-metabolizing enzymes, both rat and human hepatic subcellular fractions were used as consequent in vivo experiments in rat are being planned.

## 2. Results

In the present study, inhibitory effects of acyclic sesquiterpenes *cis*-nerolidol (CNER), *trans*-nerolidol (TNER), and farnesol (FAR), which are commonly found in the human diet, on cytosolic and microsomal biotransformation enzymes, were tested in human and rat liver subcellular fractions.

### 2.1. Screening for Enzyme Inhibition

First, the inhibitory effect of sesquiterpenes at final concentrations of 100 µM on the main hepatic biotransformation enzymes was assessed by measuring enzyme activities towards specific substrates. This high concentration was tested first to find out whether there is any inhibition at all. Specific activities of selected cytochromes P450 (CYP1A2, CYP2B, CYP3A), with and without sesquiterpenes in rat and human microsomes, are given in Table 1. Neither carbonyl-reducing enzymes, nor conjugation enzymes were inhibited by 100 µM sesquiterpenes. Specific activities of selected carbonyl-reducing enzymes (AKR1A, AKR1C, CBR1 and NQO1), and conjugation enzymes (GSTs, UGT and SULT) in rat and human liver subcellular fractions, together with these activities in the presence of 100 µM CNER, TNER and FAR, are available as supplementary files (Tables S1 and S2).

From all the tested enzymes, only CYP1A2 (EROD activity) and CYP2B/3A (BROD activity), both in human and rat microsomal fractions, were inhibited by CNER, TNER and FAR. Non-enzymatic interference of sesquiterpenes, with some compounds in the reaction mixtures (fluorescence quenching), was considered and excluded.

### 2.2. Determination of IC_50_

CYP1A2 (EROD activity) and CYP2B/3A (BROD activity) were strongly inhibited by testing sesquiterpenes in screening experiment. These enzymes were selected for further testing including determination of 50% inhibition concentration (IC_50_) in human and rat microsomes. Obtained IC_50_ values and their 95% confidence intervals are given in Table 2.

In humans, specific CYP1A2 inhibitor α-naphthoflavone (ANF) and CYP3A4 inhibitor ketoconazole (KET) were used for comparison of the inhibitory effect with sesquiterpenes. The 5 µM ANF inhibited EROD activity by 92% but 5 µM FAR, CNER and TNER, only by 77%, 50%, and 38%, respectively. On the other hand, the 5 µM KET inhibited BROD activity by 66%, while the inhibitory effect of 5 µM sesquiterpenes was stronger: FAR inhibited BROD activity by 85%, CNER by 71%, and TNER by 68%.

The inhibitory effect of CNER, TNER and FAR on other human CYP activities catalysed by CYP2A6, CYP2B6, CYP2C9, CYP2C19, CYP2D6, CYP2E1, and CYP3A4/5 and corresponding IC_50_ values were determined, as high inhibitory effect of acyclic sesquiterpenes toward EROD and BROD activities was found in screening experiment. However, no such a high inhibition was observed in other CYP activities. Data are presented in Table 3.

### 2.3. Mechanism of Enzyme Inhibition

The type of inhibition for CYP1A2 (EROD activity) and CYP2B/3A (BROD activity) by TNER was determined in human and rat microsomal fractions. When the inhibition of CYPs was tested, microsomes were incubated with different concentrations of ethoxyresorufin or benzyloxyresorufin (0.5–5.0 µM) in the presence or absence of TNER (5 µM).

**Figure 2:**
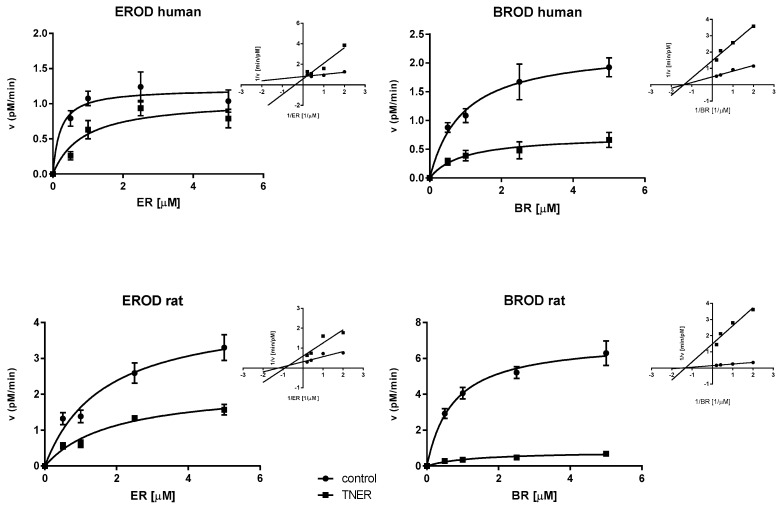
Michaelis-Menten plot of EROD and BROD activities in control and 5 µM TNER inhibited reactions. The human CYP1A2 (EROD human) was competitively inhibited and rat CYP1A2 (EROD rat) non-competitively. BROD activity was inhibited non-competitively by TNER both in human and rat. Human and rat liver microsomes were incubated with 0.5–5.0 µM ethoxyresorufin (ER) or benzyloxyresorufin (BR) in the absence (control) or presence of TNER. Lineweaver-Burke plots show the type of inhibition.

The results of kinetic study are presented in Figure 2. The human CYP1A2 was inhibited by TNER competitively, while non-competitive type of CYP1A2 inhibition by TNER was observed in rat microsomes. On the other hand, TNER acted as a non-competitive inhibitor toward human and rat BROD activity, as similar K_M_ and apparent K’_M_ values and lower apparent V´_MAX_ than V_MAX_ values were found in both species. Kinetic parameters are given in Table 4.

## 3. Discussion

Inhibition of drug-metabolizing enzymes is important from several points of view. Regarding drugs and toxic xenobiotics, the inhibition of the enzymes catalyzing their deactivation leads to augmented plasma concentration of biologically active/toxic substance. By this way, drug efficacy can be prolonged and, together with increased risk of undesired effects and the toxic effect of xenobiotics, can get worse. On the other hand, the inhibition of enzymes that activate xenobiotics into more reactive metabolites can decrease cellular toxicity of these xenobiotics and could contribute to chemo-prevention. In every way, it is necessary to know if all components of human food, as well as all potential drugs, are able to inhibit the main drug-metabolizing enzymes. For that reason, the inhibitory effects of many natural compounds, such as flavonoids, towards drug-metabolizing enzymes have been intensively studied e.g., [19,20,21,22].

Although sesquiterpenes as highly lipophilic compounds can be considered as probable inhibitors of drug-metabolizing enzymes, the information about their potential inhibitory effects is limited. Moreover, all studies have been focused only on cytochromes P450 (CYPs). When ten sesquiterpenes, isolated from the rhizomes of *Curcuma aromatica*, were evaluated for their ability to inhibit CYPs, the sesquiterpene (4*S*,5*S*)-(+)-germacrone-4,5-epoxide inhibited certain CYPs very potently with IC_50_ = 1.0 µM [23]. Sesquiterpenes zederone and germacrone moderately inhibited some CYP activities (namely, CYP2B6 and CYP3A4) in human liver microsomes [24]. Another sesquiterpene alantolactone showed a potent inhibitory effect on CYP3A4 activity with IC_50_ value of 3.6 µM in human liver microsomes [25]. Cedrol, β-cedrene and thujopsene, bioactive sesquiterpenes found in cedar essential oil, showed significant inhibition of several CYP isoforms in human liver microsomes [26].

In our study, the potential inhibitory effect of three acyclic sesquiterpenes FAR, CNER and TNER on the main drug-metabolizing enzymes was studied in rat and human hepatic subcellular fractions. In addition to CYPs, the activities of carbonyl-reducing enzymes and the main conjugation enzymes were also included in the study, with the aim to obtain more complex information. With respect to the necessity of further verifying of the results obtained in vitro in subsequent in vivo experiments, both rat and human samples were used in the present study. We aim to know if the sensitivity of drug-metabolizing enzymes to sesquiterpenes in rats is similar to those in humans, and thus rats could be a suitable experimental animal for planned evaluation of drug–sesquiterpene interactions in vivo.

Firstly, the inhibitory potency of acyclic sesquiterpenes at fixed concentration (100 µM) toward the main drug-metabolizing enzymes was screened in rat and human hepatic cytosols and microsomes. While marked inhibition of two CYP-mediated activities (EROD and BROD) by all three sesquiterpenes was observed, the activities of other enzymes were not affected by any sesquiterpene. Although the specific activities of individual enzymes mostly differed between rat and human, the effect of sesquiterpenes seemed to be similar: acyclic sesquiterpenes inhibited only EROD and BROD activities in both species.

Based on these results, the inhibitory effects of acyclic sesquiterpenes toward EROD and BROD activities were tested in details to obtain IC_50_ values, and to find out the type of inhibition. EROD activity is ascribed mainly to CYP1A2 in liver microsomes, while BROD activity mainly to CYP2B1 in rat, and to CYP3A4/2B6 activities in human [27].

Concerning BROD activities, IC_50_ values of three sesquiterpenes tested were similar (regardless of structure,) and they were slightly lower in humans (1.3–2.4 µM) than in rats (4.4–6.4 µM) microsomes. Interestingly, the inhibitory effect of acyclic sesquiterpenes was stronger than the effect of KET, a well-known CYP3A4 inhibitor. As CYP3A4 metabolizes about 50% of all drugs, its inhibition by sesquiterpenes might result in drug–sesquiterpene interactions. In the kinetic study, TNER (as a selected representative) acted as non-competitive inhibitor in both species.

In the case of EROD activity, the inhibitory potency differed more among sesquiterpenes, as well as between rat and human microsomes. Model CYP1A inhibitor ANF had a stronger effect than acyclic sesquiterpenes. TNER inhibited EROD competitively in humans but non-competitively in rats. The sesquiterpene mediated inhibition of CYP1A could represent a protective effect of sesquiterpenes, as CYP1A often catalyzes formation of toxic metabolites from environmental pollutants.

Consequently, the inhibitory effects of sesquiterpenes were followed up in other CYP-mediated activities in human liver microsomes. Nine substrates relatively specific for CYP2A6, CYP2B6, CYP2C9, CYP2C19, CYP2D6, CYP2E1, and CYP3A4/5 were used, and corresponding IC_50_ values were determined. However, none or weak inhibition by acyclic sesquiterpenes have been observed. Surprisingly, hydroxylation of midazolam and testosterone, both ascribed to CYP3A enzymes, were inhibited only mildly, although BROD activity (also ascribed to CYP3A4) was strongly inhibited by all sesquiterpenes tested. TNER was the most potent inhibitor of testosterone hydroxylation with IC_50_ 51 µM, while CNER was the most effective toward midazolam hydroxylation with IC_50_ 66 µM. The discrepancy of these results can be explained by different substrate concentrations used in the enzyme assays (5 µM benzyloxyresorufin, 100 µM testosterone), and by different binding sites of these substrates [28].

Until now, the inhibitory effect of FAR on CYPs was tested only in rabbit liver microsomes. The obtained results showed that ethoxycoumarin deethylase and diclofenac-4-hydroxylase activities were most sensitive to FAR, while caffeine-8-hydroxylation and taxol-6-hydroxylation were not inhibited at all. The observed inhibition appeared to be reversible, and was not strictly competitive, but rather mixed in nature [29]. Concerning TNER and CNER, no information about their inhibitory effects toward drug-metabolizing enzymes has been reported yet.

## 4. Materials and Methods

### 4.1. Chemicals and Reagents

Sesquiterpenes *cis*-nerolidol (CNER), *trans*-nerolidol (TNER), farnesol (FAR), and other chemicals benzyloxyresorufin, ethoxyresorufin, resorufin, coumarin, 7-ethoxy-4-(trifluoromethyl)coumarin, *S*-mephenytoin, diclofenac, bufuralol, testosterone, midazolam, chlorzoxazone, menadione, 1-chloro-2,4-dinitrobenzene, p-nitrophenol, p-nitrophenylsulphate, 2-napthol, 3’-phosphoadenosine-5’-phosphate, acenaphthenol, reduced glutathione, UDP-glucuronic acid, cytochrome c, NADH, NADP and NADPH, were purchased from Sigma-Aldrich (Prague, Czech Republic). All other chemicals used were of HPLC or analytical grade.

### 4.2. Preparation of Liver Microsomal and Cytosolic Fractions

Male Wistar rats (10–12 weeks old) were obtained from Velaz (Prague-Lysolaje, Czech Republic). They were housed in air-conditioned animal quarters with a 12 h light/dark cycle. Food (a standard rat chow diet) and water were provided ad libitum. The animal protocols used in this work were evaluated and approved by the Ethic Committee of the Ministry of Education, Youth and Sports (Protocol MSMT-24185/2015-11). The protocols were in accordance with the Guide for the Care and Use of Laboratory Animals (Protection of Animals from Cruelty Act No. 246/92, Czech Republic).

Human liver samples were obtained from livers excluded from transplantation for medical reasons (Cadaver Donor Programme of Transplant Centre of the Faculty of Medicine, Charles University, Hradec Králové, Czech Republic) and were used in compliance with National Ethic Laws.

Frozen liver (both rat and human) was thawed at room temperature for up to 15 min and processed to microsomal and cytosolic fractions. Briefly, livers were homogenized in a 0.1 M sodium phosphate buffer, pH 7.4, at the ratio of 1:6 (*v*/*v*), using a Potter-Elvehjem homogenizer and sonication with Sonopolus (Bandelin, Germany). The subcellular fractions were isolated by differential centrifugation of the tissue homogenate [30] and stored at −80 °C.

Protein concentrations were assayed using the bicinchoninic acid (BCA) assay according to manufacturer’s instructions (Sigma-Aldrich). Human liver microsomal fraction contains 8.1 mg of protein in 1 mL, and cytosolic fraction 12.1 mg of protein in 1 mL. Rat liver microsomal fraction contains 8.5 mg of protein in 1 ml, and cytosolic fraction 13.1 mg of protein in 1 mL.

### 4.3. Enzyme Assays

Enzyme activities were assayed in human and rat liver cytosolic and microsomal fractions. The enzyme assays (each performed in 4–8 replicates) were repeated three times (HPLC measurement of CYP activities was repeated only twice, Table 3). Inhibition studies were carried out by incubation of specific substrates in the presence of sesquiterpenes (in the range 0–100 µM). The amount of organic solvents in the final reaction mixtures did not exceed 1% (*v*/*v*).

Enzyme activities of individual CYP forms were measured according to established protocols [31]. The following microsomal CYP activities were tested: CYP2A6, coumarin 7-hydroxylation [32,33]; CYP2B6, 7-ethoxy-4-(trifluoromethyl)coumarin 7-deethylation [33,34], CYP2C9, diclofenac 4′-hydroxylation [35], CYP2C19 assay, *S*-mephenytoin 4′-hydroxylation [36], CYP2D6 on bufuralol 1′-hydroxylation [37], CYP2E1, chlorzoxazone 6-hydroxylation [38] and CYP3A4, testosterone 6β-hydroxylation [39], and midazolam 1′-hydroxylation [40,41]. Activities were measured using the Prominence HPLC system (Shimadzu, Kyoto, Japan), equipped with a LiChroCART 250-4 LiChrospher 100 RP-18 column or Chromolith^®^HighResolution RP-18 end-capped column (Merck, Darmstadt, Germany) and UV or fluorescence detection, according to the cited literature. Further incubation details are described either in the corresponding references, as well as in the papers from this laboratory e.g., [42,43,44]. CYP1A2, 7-ethoxyresorufin O-deethylation, and CYP3A4/2B6 (human), CYP2B1 (rat), benzyloxyresorufin O-debenzylation were measured using the microplate reader Tecan Infinite M200 (Tecan Group; Mannedorf, Switzerland).

The assays of all other enzymes were based on spectrophotometric detection of product formed or detection of decreasing substrate/cofactor levels using microplate reader Tecan Infinite M200 in cytosolic or microsomal fractions. Enzyme activities were determined using slight modifications of previously-published methods: carbonyl reducing enzymes (AKR1A, AKR1C, CBR1 [45], NQO1 [46]), UDP-glucuronosyltransferase (UGT [47]), sulfotransferase (SULT [48]), and cytosolic glutathione-*S*-transferase (GST [49]).

### 4.4. Screening for Enzyme Inhibition

Sesquiterpenes were first screened for inhibition of biotransformation enzymes in subcellular fractions in vitro, then the half-maximal inhibitory concentration (IC_50_) value and the mechanism of enzyme inhibition was revealed. Sesquiterpenes CNER, TNER and FAR, non-volatile liquids, were pipetted, and proper concentration was calculated according to density. All compounds were dissolved in DMSO. The final solvent concentration in the inhibition assays was 1%, which had no effect on the enzyme activity (data not shown). Non-enzymatic interference of sesquiterpenes with some compounds in the reaction mixtures (e.g., fluorescence enhancing or quenching) was considered and excluded. All experiments were performed in three replicates, if not stated otherwise.

CNER, TNER and FAR at a fixed concentration of 100 µM were screened for inhibition of cytosolic AKR1A1, AKR1C, CBR1, NQO1, SULT, and GST, and microsomal CYPs and UGT. The degree of inhibition caused by sesquiterpenes was compared with non-inhibited control reactions, which contained solvent without sesquiterpenes.

### 4.5. Determination of IC_50_

The concentration of sesquiterpenes required for 50% inhibition of enzyme activity, the IC_50_ value, was determined. Their final concentrations in the reaction mixture were proposed to cover the entire inhibitory range of these compounds (1–100 µM). The IC_50_ values for each sesquiterpene were determined by plotting sigmoidal dose response curves of specific enzyme activity vs. logarithm of sesquiterpene concentration using GraphPad Prism version 6.07 for Windows (GraphPad Software Inc., La Jolla, CA, USA).

### 4.6. Mechanism of Enzyme Inhibition

The kinetic inactivation study of the CYP1A2 (EROD activity) and CYP2B/3A (BROD activity), both in rat and human, with TNER, was performed to find out the mechanism of enzyme inhibition by this sesquiterpene. Mechanism of EROD and BROD inhibition was tested at the final ethoxyresorufin or benzyloxyresorufin concentrations 0.5–5.0 µM. The concentration of TNER was 5.0 µM.

Experimental data were fitted to the Michaelis-Menten equation, basic kinetic parameters were determined using the program GraphPad Prism version 6.07 for Windows. The type of inhibition was estimated using a Lineweaver-Burk (double-reciprocal) plot.

### 4.7. Statistical Analysis

All experiments (each sample performed in four replicates) were repeated three times (HPLC measurement of CYP activities only twice). All calculations were done using Microsoft Excel and GraphPad Prism 6.07. Statistical significance was tested by one-way Anova, and differences were considered statistically significant when *p* < 0.05. Data are presented as the mean ± standard deviation.

## 5. Conclusions

Taken together, acyclic sesquiterpenes FAR, CNER, and TNER showed certain potency to inhibit CYP1A, CYP2B and CYP3A activities in liver. On the other hand, these sesquiterpenes did not seriously affect other CYPs, carbonyl-reducing enzymes and conjugation enzymes. As CYP3A4 metabolizes about 50% of all drugs, its inhibition by sesquiterpenes might result in drug—sesquiterpene interactions. Nevertheless, the results obtained in vitro should be verified in vivo. Rat seems to be suitable model for these in vivo studies, as comparable sensitivity of drug-metabolizing enzymes to these sesquiterpenes have been observed in rat and human samples.

## Figures and Tables

**Table 1:** Specific activities of CYP1A2 (EROD activity) and CYP2B/3A (BROD activity) in human and rat liver subcellular fractions—controls and samples in the presence of 100 µM sesquiterpenes CNER, TNER and FAR.

		Specific Activity (pmol/mg/min)			
		Control	CNER	TNER	FAR
Human	CYP1A2	1.88 ± 0.19	0.163 ± 0.005 *	0.287 ± 0.013 *	0.083 ± 0.005 *
	CYP2B/3A	1.22 ± 0.05	0.168 ± 0.007 *	0.188 ± 0.005 *	0.033 ± 0.004 *
Rat	CYP1A2	22.8 ± 0.1	2.16 ± 0.04 *	1.53 ± 0.03 *	1.55 ± 0.02 *
	CYP2B/3A	1.74 ± 0.02	0.331 ± 0.009 *	0.153 ± 0.008 *	0.212 ± 0.003 *

Mean ± S.D., *n* = 3; * significantly lower (*p* < 0.05) comparing to control.

**Table 2:** Inhibition concentration (IC_50_) of sesquiterpenes CNER, TNER, FAR, and of specific inhibitors ANF (CYP1A2) and KET (CYP2B/3A) to CYP1A2 (EROD activity) and CYP2B/3A (BROD activity) in human and rat liver microsomes.

			CNER	TNER	FAR	ANF/KET
Human	CYP1A2	IC_50_ (µM)	2.49	8.69	1.83	0.41
		95% CI	1.38 to 4.48	5.96 to 12.7	1.39 to 2.41	0.22 to 0.75
	CYP2B/3A	IC_50_ (µM)	1.32	2.40	1.78	2.68
		95% CI	1.02 to 1.71	2.02 to 2.83	1.59 to 2.01	2.07 to 3.48
Rat	CYP1A2	IC_50_ (µM)	5.70	4.38	16.1	-
		95% CI	5.30 to 6.14	3.91 to 4.92	14.0 to 18.5	-
	CYP2B/3A	IC_50_ (µM)	6.47	5.11	4.43	-
		95% CI	6.07 to 6.89	4.34 to 6.02	3.82 to 5.14	-

Mean ± S.D., *n* = 3; 95% CI = 95% confidence intervals.

**Table 3:** Inhibition concentration (IC_50_) of sesquiterpenes CNER, TNER and FAR to CYP2A6, CYP2B6, CYP2C9, CYP2C19, CYP2D6, CYP2E1, and CYP3A4/5 in human liver microsomes.

CYP	Substrate	IC_50_ (µM)		
		CNER	TNER	FAR
CYP2A6	coumarin	-	-	85.9 ± 22.9
CYP2B6	7-ethoxy-4-(trifluoromethyl)coumarin	-	-	-
CYP2C9	diclofenac	-	-	-
CYP2C19	S-mephenytion	-	76.4 ± 52.1	-
CYP2D6	bufuralol	-	-	-
CYP2E1	chlorzoxazone	-	-	-
CYP3A4/5	testosterone	186 ± 125	50.5 ± 13.5	-
CYP3A4/5	midazolam	66.1 ± 30.5	-	-

Mean ± S.D., *n* = 2.

**Table 4:** Kinetic parameters obtained for CYP1A2 (EROD activity) after incubation of human and rat liver microsomes with ethoxyresorufin (0.5–5.0 µM) in the presence (K’_M_, V’_MAX_) or absence (K_M_, V_MAX_) of TNER (5 µM), and for CYP2B/3A (BROD activity) after incubation of human and rat liver microsomes, with benzyloxyresorufin (0.5–5.0 µM) in the presence (K’_M_, V’_MAX_) or absence (K_M_, V_MAX_) of TNER (5 µM).

	Enzyme	K_M_ (µM)	K’_M_ (µM)	V_MAX_ (nM/min)	V’_MAX_ (nM/min)	Ki (µM)
Human	CYP1A2	0.21 ± 0.11	0.87 ± 0.41	1.21 ± 0.10	1.06 ± 0.16	0.92 ± 0.52
	CYP2B/3A	0.93 ± 0.22	0.94 ± 0.39	2.27 ± 0.17	0.74 ± 0.10	2.41 ± 0.33
Rat	CYP1A2	1.64 ± 0.45	1.89 ± 0.49	4.32 ± 0.47	2.21 ± 0.24	4.68 ± 0.66
	CYP2B/3A	0.74 ± 0.12	1.26 ± 0.39	7.04 ± 0.35	0.81 ± 0.09	0.55 ± 0.11

## Supplementary Materials

Supplementary materials are available online.
