# Supplementary material for: Nerolidol and Farnesol Inhibit Some Cytochrome P450 Activities but Did Not Affect Other Xenobiotic-Metabolizing Enzymes in Rat and Human Hepatic Subcellular Fractions
Source: Molecules. 2017 Mar 24;22(4):509. doi: 10.3390/molecules22040509 (PMC6154719; doi:10.3390/molecules22040509)
Supplement: Supplementary file 1 [file molecules-22-00509-s001.docx]

**Supplementary material**

**Table S1.** Specific activities of carbonyl-reducing enzymes in human and rat liver subcellular fractions – controls and samples in the presence of 100 µM sesquiterpenes CNER, TNER and FAR.

|  |  | **Specific activity [nmol/mg/min]** | | | |
| --- | --- | --- | --- | --- | --- |
|  |  | **Control** | **CNER** | **TNER** | **FAR** |
| Human | AKR1A | 8.45 ± 0.90 | 6.88 ± 0.29 | 6.75 ± 0.22 | 7.62 ± 0.30 |
|  | AKR1C | 23.5 ± 2.4 | 22.7 ± 1.5 | 21.4 ± 1.0 | 24.0 ± 1.5 |
|  | CBR1 | 6.62 ± 1.84 | 7.17 ± 0.56 | 5.67 ± 0.67 | 6.83 ± 0.70 |
|  | NQO1 | 6.38 ± 0.34 | 5.44 ± 0.82 | 5.53 ± 0.51 | 5.23 ± 1.03 |
| Rat | AKR1A | 3.60 ± 0.13 | 3.86 ± 0.47 | 3.71 ± 0.56 | 3.48 ± 0.32 |
|  | AKR1C | 14.8 ± 1.8 | 14.6 ± 2.1 | 14.8 ± 1.6 | 14.9 ± 1.5 |
|  | CBR1 | 21.7 ± 1.6 | 19.5 ± 1.4 | 20.5 ± 1.6 | 21.1 ± 1.4 |
|  | NQO1 | 25.3 ± 5.6 | 21.7 ± 3.6 | 21.8 ± 2.4 | 22.2 ± 3.3 |

Mean ± S.D., n = 3
Carbonyl reductase 1 (CBR1), NADPH-quinone oxidoreductase 1 (NQO1), aldo-keto reductase (AKR).

**Table S2.** Specific activities of conjugation enzymes in human and rat liver subcellular fractions – controls and samples in the presence of 100 µM sesquiterpenes CNER, TNER and FAR.

|  |  | **Specific activity [nmol/mg/min]** | | | |
| --- | --- | --- | --- | --- | --- |
|  |  | **Control** | **CNER** | **TNER** | **FAR** |
| Human | GST | 223 ± 19 | 209 ± 10 | 235 ± 13 | 181 ± 13 |
|  | SULT | 2.73 ± 0.18 | 3.12 ± 0.29 | 3.20 ± 0.26 | 2.72 ± 0.23 |
|  | UGT | 7.12 ± 0.35 | 7.09 ± 0.34 | 7.08 ± 0.39 | 7.49 ± 1.08 |
| Rat | GST | 350 ± 8 | 334 ± 22 | 354 ± 37 | 345 ± 20 |
|  | SULT | 8.74 ± 0.52 | 8.74 ± 0.26 | 8.48 ± 0.28 | 8.55 ± 0.30 |
|  | UGT | 16.2 ± 0.98 | 15.9 ± 1.11 | 16.4 ± 2.46 | 14.3 ± 3.15 |

Mean ± S.D., n = 3
glutathione S-transferase (GST), sulfotransferase (SULT), UDP-glucuronosyltransferase (UGT)
